# Extraction and Chromatographic Approaches for Coumarin, Furocoumarin, and Polymethoxyflavone Characterization in Foods

**DOI:** 10.3390/foods13162517

**Published:** 2024-08-12

**Authors:** Giovanna Cafeo, Elisa Irrera, Marina Russo, Paola Dugo

**Affiliations:** 1 Messina Institute of Technology c/o Department of Chemical, Biological, Pharmaceutical and Environmental Sciences, Former Veterinary School, University of Messina, Viale G. Palatucci snc, 98168 Messina, Italy; giovanna.cafeo@studenti.unime.it (G.C.); elisa.irrera@studenti.unime.it (E.I.); paola.dugo@unime.it (P.D.); 2 Chromaleont s.r.l., c/o Messina Institute of Technology c/o Department of Chemical, Biological, Pharmaceutical and Environmental Sciences, Former Veterinary School, University of Messina, Viale G. Palatucci snc, 98168 Messina, Italy

**Keywords:** coumarin, furocoumarins, *Citrus*, cinnamon, oxygen heterocyclic compounds extraction, oxygen heterocyclic compounds analysis

## Abstract

Oxygen heterocyclic compounds play a beneficial role in plants, and their presence in foods, such as *Citrus* fruits, cinnamon, carrots, and parsley, has been documented in recent years. Published research articles reported several extractions and chromatographic techniques for their determination. The aim of this review was to take into consideration the research articles published from 2016 to 2024 in which the authors developed extraction and chromatographic analysis methods of oxygen heterocyclic compounds in foods. The objective of this review was to assist researchers in choosing the best approach for their future work by identifying all the possible approaches to characterize coumarins, furocoumarins, and polymethoxyflavones in foodstuffs.

## 1. Introduction

**Figure 1:**
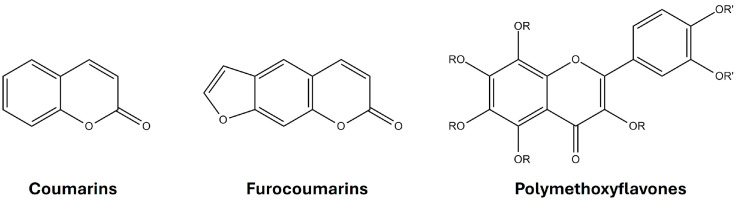
Chemical structure of coumarins, furocoumarins, and polymethoxyflavones.

Oxygen heterocyclic compounds (OHCs), which include coumarins, furocoumarins, and polymethoxyflavones (Figure 1), are secondary plant metabolites. These compounds play a beneficial role in plants by providing protection against infections and supporting growth. Their presence in foods has been extensively documented in recent years by researchers [1,2,3,4]. These compounds are primarily found in *Citrus* fruits, cinnamon, carrots, parsley, and similar foods.

While the positive effects of polymethoxyflavones on human health are well-documented [5,6,7], there are some concerns about furocoumarins and coumarin. International authorities, such as the European Parliament and the International Fragrance Association (IFRA), have issued amendments regarding the concentration of coumarin and furocoumarins in foods and cosmetic products to protect human health [8,9]. Negative health effects have been observed following the ingestion of coumarin [10,11]. According to the European Food Safety Authority (EFSA) in 2004 [12], and the European Parliament in 2008 [13], the tolerable daily intake of coumarin is 0.1 mg per kg of body weight; for this reason, a maximum level permitted of coumarin in several foods, such as desserts, bakery products, and cereals, is regulated [2,14]. No specific recommendations were provided for the daily intake of other oxygen heterocyclic compounds in foodstuffs. On the other hand, regulations limiting the presence of these molecules are particularly stringent for cosmetic products because of the harmful interaction between furocoumarins and ultraviolet A rays [15,16].

Numerous studies have investigated the presence of oxygen heterocyclic compounds in foods, employing various extraction techniques such as liquid–liquid extraction (LLE), solid–liquid extraction (SLE), microwave and/or ultrasound-assisted extraction (UAE), solid-phase extraction (SPE), and Quick, Easy, Cheap, Effective, Rugged, Safe (QuEChERS) [1,2,3,6,17]. These extraction methods are followed by chromatographic analyses for coumarin, furocoumarin, and polymethoxyflavone characterization. Most studies focus on the analysis of coumarins in foods, utilizing different chromatographic techniques like high-performance liquid chromatography (HPLC) [18,19], supercritical fluid chromatography (SFC) [20,21,22], and thin-layer chromatography (TLC) [23,24,25] for their determination. But it is now well known that the most suitable analytical technique for oxygen heterocyclic compound determination is HPLC coupled with both spectrophotometric (PDA) and mass spectrometer (MS) detectors [4,26,27].

When assessing an analytical method that includes both an extraction step and chromatographic analysis, it is crucial to consider parameters like recovery, limit of detection (LoD), limit of quantification (LoQ), precision, and accuracy. Additionally, attention to environmental impact and operator safety is significant. Consequently, factors such as timing, the nature of reagents used, the amount of waste generated, and the energy consumed are important considerations for prioritizing environmental protection [28].

The aim of this review was to take into consideration the research articles published in the last eight years in which the authors developed extraction and chromatographic analysis methods of oxygen heterocyclic compounds in foods. The objective was to compare all the proposed methods for all oxygen heterocyclic classes in terms of the use of traditional or innovative methodology, using conventional or more advanced extraction/analytical techniques, and to determine if an eco-friendly solution could be the best way to investigate these molecules.

### Paper Selection

In order to be able to draft this review, a bibliographic search was carried out using Scopus and Google Scholar. These keywords were used “oxygen heterocyclic compounds”, “coumarins”, “extraction”, “furocoumarins”, “polymethoxyflavones”, “analysis”, and “chromatography”. From this research, more than 5000 reviews and research articles were found. After a careful and accurate selection of this first investigation, sixteen reviews and fifty-six research articles focused only on foodstuffs (beverages, snacks, extra-virgin olive oil, essential oils, fruits, vegetables, jams, and so on) were selected. The other articles were not considered because they did not deal with foodstuffs, but rather with traditional Chinese medicine, or they focused on the use of biological assays that did not match the object of this review.

## 2. Extraction Methods

**Figure 2:**
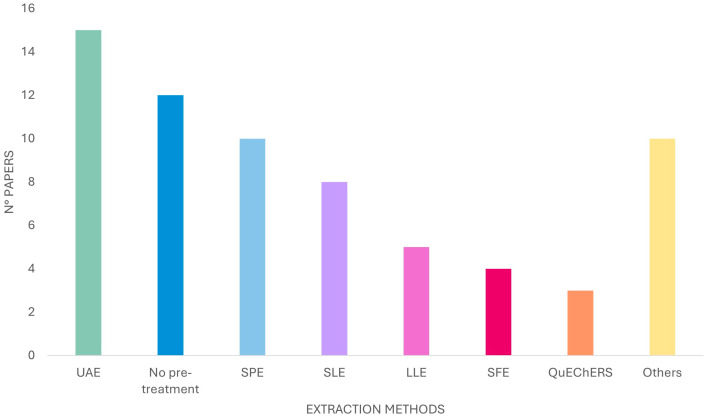
Types of sample pre-treatments used for OHC extraction from foodstuffs (from 2016 to 2024).

For OHCs investigation in foodstuffs, in most cases, an extraction step is mandatory. Based on a literature survey, (a) UAE, (b) SLE, (c) LLE, (d) SPE, (e) QuEChERS, and (f) supercritical fluid (SFE) extraction techniques were the most employed methods (see Figure 2). It is notable that only for liquid samples, some authors reported only a simple pre-treatment step of the investigated food before the analytical process. For example, a filtration on a 0.45 μm filter was used to characterize coumarins in a cachaça sample [29]. Only one dilution step was used to determine OHCs in cold-pressed *Citrus* essential oils, *Citrus*-flavored extra-virgin olive oils, wines, *Citrus* juices [30,31,32,33,34,35,36,37,38,39,40].

### 2.1. UAE

The literature survey revealed that ultrasound-assisted extraction is the prevalent method employed to enhance the extraction of OHCs from plant materials and foodstuffs, as reported in Figure 2. Over the years, various solvents with different polarities have been tested in order to identify the most suitable solvent for the aforementioned molecules. In particular, methanol and ethanol, both pure and in several dilutions, as well as ethyl acetate, have emerged as the most commonly used solvents.

It is well known that cinnamon is the richest source of coumarin in the human diet, so several research articles proposed an extraction procedure from cinnamon bark and flavoring powder, as well as beverages and foods flavored with cinnamon [41,42,43,44]. Considering the procedures adopted, methanol appears to be the solvent of choice. Differences among the validated methodologies were related to extraction time and the sample/solvent ratio. Firstly, Solaiman et al. [41] presented coumarin extraction from 500 mg of cinnamon bark previously pulverized with 25 mL of a methanol/water solution (80:20, *v*/*v*) with 60 min as total extraction time. Three years later, Cao et al. [42] used the same solvent mixture (250 mL) to extract coumarin from 500 mg of cinnamon flavoring powder but with a shorter extraction time (20 min). Recently, Pages-Rebull and colleagues [43] reported a faster procedure (15 min) to recover the coumarin from cinnamon using only methanol (2 mL for 500 mg of sample). On the other hand, a longer extraction time (one hour) was employed by Kruger et al. [44] to extract the coumarin from 500 mg of cinnamon with 100 mL of pure methanol. The same authors investigated extraction from foodstuffs but with a different sample/solvent ratio, including samples of tea (500 mg/10 mL), breakfast cereal and milk rice (500 mg/2 mL), and cinnamon buns (500 mg/5 mL).

The coumarin content was also investigated in *Citrus* fruits and *Citrus*-flavored foodstuffs [45,46]. Aznar et al. [45] proposed an extraction procedure for 1 g of dried and powdered finger lime peels and pulps with 60 mL of 80% aqueous methanol for 90 min of sonication. Ethyl acetate was chosen as the extraction solvent by Cafeo and co-workers [46] to extract coumarin and twenty-seven other OHCs (reported in Table 1) in *Citrus*-flavored jams and bakery products as follows: 1 g of sample extracted with 3 mL of solvent for 45 min.

Furocoumarins, coumarins, and polymethoxyflavones were recovered from *Citrus* fruits and *Citrus*-flavored foodstuffs [47,48,49] using both methanol and ethyl acetate as extraction solvents. Arigò et al. [47] chose ethyl acetate to carry out a solid–liquid extraction of 35 OHCs (reported in Table 1) from lemon marmalade. In their study, 10 g of a sample was sonicated with 30 mL of solvent for 45 min. Zhao et al. [48] and Guo et al. [49] selected methanol as an extraction solvent and recovered twenty and twenty-eight OHCs from pummelo fruit and Satsuma mandarin peels and pulps, respectively, in 90 min. Zhao and co-workers [48] used 400 mg of the sample with 25 mL of methanol, while Guo et al. [49] used 5 g of each sample with 45 mL of solvent.

Coumarins and furocoumarins were also extracted from some herbaceous materials. In 2020, Fu et al. [50] extracted scopolin and scopoletin from Artemisia annua herbal samples with 20 mL of 80% ethanol using a sample–solvent ratio of 1 to 40 and a total extraction time of 1 h. On the other hand, Dresler et al. [51] extracted six coumarins and six furocoumarins from the herb *Heracleum sphondylium* L. and the cortex of *Aesculus hippocastanum* L. using just a tenfold amount of 80% methanol with respect to the ground sample/powder and in half the time reported by Fu et al. [50].

### 2.2. SLE

To date, solid–liquid extraction is a widespread technique for solid sample preparation, based on analyte partitioning between the matrix and the extraction solvent [52]. Methanol, ethanol, and water were the most commonly used extraction solvents in the studies reviewed in this paper. To improve the extraction efficiency of this technique, several strategies were considered, including the use of a mechanical shaker. Firstly, in 2017, Hyun et al. [53] stirred Shiranuhi [(*Citrus unshiu* Marc. × *C. sinensis* Osbeck) × *C. Reticulata* Blanco)] fruit peels three times with 80% ethanol for 24 h to extract tetra-O-methyl-scutellarein. The same year, Machynakova et al. [54] recovered nine coumarins, as reported in Table 1, from the aerial parts of *Melilotus officinalis* L. (Meliloti herba) and propolis. In their study, 5 g of Meliloti herba powder was stirred with 30 mL of distilled water for 60 min, while 1 g of crude propolis was stirred with 40 mL of ethanol for 72 h. The next year, Hrobonova and co-workers [55] extracted coumarin, 4-hydroxycoumarin, and dicoumarol from the aerial parts of sweet clover (*Melilotus officinalis* L.) herb and hay samples. A total of 0.1 g of each sample was stirred on a mechanical shaker for 60 min with 20 mL of methanol.

Fayek et al. [56] extracted nobiletin from grapefruit, lime, sweet orange, and mandarin peels with a multi-step extraction. In their method, 600 mL of 80% methanol was percolated through 200 g of fresh peels, the resulting extract was evaporated, and 10 g of the residue was suspended in 30 mL of distilled water and then extracted with n-hexane. Two years later, the same authors [57] extracted twenty-one OHCs, as reported in Table 1, from the peels of four *Citrus* species [*C. reticulata* Blanco cv. Egyptian, *C. sinensis* (L.) Osbeck cv. Olinda Valencia, *C. aurantiifolia* Swingle cv. Mexican and *C. paradisi* Macfad. cv. Duncan]. They homogenized 160 mg of *Citrus* peel powder with 7 mL of 100% methanol using a Turrax mixer for 20 s for five periods. The obtained extract was purified with a C18 cartridge, and the analytes were eluted with 6 mL of methanol.

A longer extraction procedure was used by Moreno-Ley et al. [58]. In their method, six vanilla samples were incubated with a mixture of ethanol–water at 65% for three months in darkness with a sample–solvent ratio of 1 to 1 to extract coumarin. Lastly, in 2020, Aboul Naser et al. [59] extracted six polymethoxyflavones (see Table 1) from sweet orange (*Citrus sinensis*) peel powder (250 g) with 2 L of petroleum ether repeated three times.

### 2.3. LLE

LLE extraction is limited to liquid food samples, namely, beverages. Four research articles on OHCs recovery were considered [46,47,60,61]. The alcoholic and non-alcoholic beverages investigated included the following: 16 citrus-flavored beers [60], infusion and liquors [46,47], citrus juices, and cinnamon-flavored liquor [46]. The extraction procedure was carried out with ethyl acetate, in particular, 10 mL of each sample was extracted three times with 10 mL of solvent by manually shaking in a separatory funnel. For the list of compounds extracted, see Table 1. Recently, Cafeo et al. [61] developed a miniaturized extraction procedure and extracted 36 OHCs from 1 g of citrus and herb liquors with 1 mL of ethyl acetate.

### 2.4. SPE

Food samples can be considered complex mixtures that necessitate the use of specific sample pre-treatment methods, such as the use of SPE cartridges, in order to clean and/or concentrate analytes prior to chromatographic analysis. Several packing materials were selected to purify OHCs from complex food matrices.

In their first study, Li and co-workers used a C-18 SPE cartridge [62] and then, two years later, a C-8 [63] SPE cartridge to extract OHCs from citrus juices. After SPE cartridge conditioning, the molecules of interest were eluted with 5 mL ethyl acetate. A C-18 SPE cartridge was also used by Wang et al. [64] to enrich the polymethoxyflavone fraction (nobiletin, tangeretin, and 5-demethylnobiletin) obtained from the ultrasonic extraction of Ougan (Citrus reticulata cv. Suavissima) peels. Methanol was selected as the eluent.

Hrobonova et al. used a lab-made molecularly imprinted polymer (MIP)-based sorbent on the surface of magnetic particles for solid phase extraction [65,66]. The authors purified coumarins (see Table 1) from *Melilotus officinalis* L. [65] and wines [66] with a laboratory made SPE cartridge packed with MIP material, using 2 mL of methanol/acetic acid (9/1, *v*/*v*) as the eluent. MIP material was also used by Machynakova et al. [67] and Nie et al. [68] to carry out a magnetic solid-phase extraction of coumarins from food samples (cinnamon sticks, ground cinnamon, cinnamon cereals, dried archangel, chamomile, lavender, soft drink, biscuit, and sesame paste). Each sample was subjected to solvent treatment (water [67] and acetonitrile [68]) prior to the multi-step magnetic solid phase extraction procedure.

In 2022, Kalogiuri et al. [69] proposed a novel capsule-phase microextraction (CPME) method for coumarin isolation from bakery products, namely, Greek tsoureki, cinnamon biscuits, and Italian panettone. The extract obtained with methanol–water (80:20, *v*/*v*) was subjected to a clean-up procedure through a sol-gel C18 CPME device under magnetic stirring for 20 min. Analyte elution was obtained using 3 mL of methanol under magnetic stirring for 15 min.

### 2.5. QuEChERS

Some procedures for coumarin and furocoumarin extraction employed the QuEChERS methodology. QuEChERS powder (magnesium sulfate/sodium acetate) was used to extract furocoumarins from Ruby red grapefruit (whole, flesh, peel, and juice) [70] and other popularly consumed foods and beverages in the United States [71]. For both applications, 5 g of each sample was extracted with 10 mL of acetonitrile. Vetter et al. [72] used a QuEChERS powder (magnesium sulfate/sodium chloride mixture (4:1)) and10 g of cinnamon-flavored bakery products to extract coumarin with 20 mL of acetonitrile/water (1:1, *v*/*v*).

### 2.6. SFE

SFE has emerged as a promising alternative technique to conventional solvent extraction for OHC isolation from foods [73]. CO_2_ is the extraction agent of choice because of its environmental friendliness. However, in some instances, the addition of modifiers or cosolvents is necessary to enhance the extraction of polar compounds, and methanol and ethanol are commonly used for this purpose [74].

Oba and coworkers [75] carried out SFE extraction of nobiletin from Citrus Unshiu peels. Conventional supercritical CO_2_ extraction was compared with the addition of a modifier, namely, ethanol, mixed with CO_2_ before the extraction cell. The impact of varying ethanol concentrations on the yield of nobiletin was investigated. The results demonstrated that an increase in ethanol concentration in supercritical CO_2_ led to an enhancement in nobiletin yield. Long et al. [76] employed preparative SFE to enrich the extract of three polymethoxyflavones, namely, 3,5,6,7,8,3′,4′-heptamethoxyflavone, nobiletin, and tangeretin, from Citri reticulatae pericarpium (CRP). Jokic et al. [73] used supercritical CO_2_ in dynamic extraction mode to extract scopoletin from Helichrysum italicum (Roth) G. Don fil. ssp. italicum flowers with an extraction run of 90 min. Two operating parameters, namely, pressure and temperature, were varied, and it was found that 20 MPa and 40 °C were the conditions that led to the highest yield (6.31%) with the highest content of scopoletin (1.933 mg/100 g).

### 2.7. Miscellanea

Katekhaye et al. [77] evaluated the efficiency of several methods (microwave-assisted (MAE), heat reflux, maceration, Soxhlet, and ultrasonic-assisted) in extracting bergapten from *Pithecellobium dulce* bark. For each extraction methodology tested, the authors used 2 g of dried sample and 40 mL of chloroform, except for the Soxhlet extraction, which was carried out with 80 mL of chloroform and an extraction time from 10 min (MAE) to 24 h (maceration). The outcomes demonstrated that MAE exhibited a higher extraction yield that consumed a reduced amount of solvent and required the shortest extraction time.

Ananthakishnan et al. [78] extracted coumarin from 10 samples *Cinnamomum verum* barks (1 g each sample) with methanol for 3 h in a Soxhlet apparatus.

**Table 1:** List of the research papers that investigated both the extraction and the analysis of OHCs in foodstuffs (from 2016 to 2024).

Samples	OHCs	Extraction	Method	Ref.
Cachaças	**Two Cs**: 4-methylumbelliferone, coumarin	Filtration	HPLC-DAD	[29]
Wines and spirits	**Six Cs**: esculetin, scopoletin, fraxetin, umbelliferone, 4-methylumbelliferone, coumarin	Dilution	HPLC-HRMS	[30]
*Citrus* essential oils	**Fifteen FCs**: psoralen, bergapten, xanthotoxin, isopimpinellin, oxypeucedanin, oxypeucedanin hydrate, byakangelicol, byakangelicin, heraclenin, 8-geranyloxypsoralen, bergamottin, imperatorin, isoimperatorin, phellopterin, and epoxybergamottin	Dilution	HPTLC	[31]
Cold-pressed *Citrus* essential oils	**Ten Cs**: aurapten, citropten, epoxyaurapten, herniarin, isomerazin, meranzin, meranzin hydrate, 5-geranyloxy-7-methoxycoumarin, 5-isopentenyloxy-7-methoxycoumarin, osthol; **15 FCs**: bergamottin, bergapten, byakangelicin, byakangelicol, cnidicin, cnidilin, epoxybergamottin, isoimperatorin, isopimpinellin, oxypeucedanin, oxypeucedanin hydrate, phellopterin, 8-geranyloxypsoralen, epoxybergamottin hydrate; **5 PMFs**: nobiletin, sinensetin, tangeretin, tetra-O-methylscutellarein, heptamethoxyflavone	Dilution	SFC-QqQ- MS/MS	[32]
Cold-pressed *Citrus* essential oils, *Citrus*-flavoured juices and beverages	**Eigth Cs**: coumarin, herniarin, meranzin, meranzin hydrate, citropten, epoxyaurapten, auraptene, 5-geranyloxy-7-methoxy-coumarin; **21 FCs**: 8-methoxypsoralen, byakangelicin, psoralen, oxypeucedanin hydrate, angelicin, isopimpinellin, heraclenin, oxypeucedanin, bergapten, byakangelicol, isobergapten, 6′,7′-dihydroxybergamottin, imperatorin, trioxalen, phellopterin, cnidilin, epoxybergamottin, isoimperatorin, cnidicin, 8-geranyloxypsoralen, bergamottin; **seven PMFs**: sinensetin, nobiletin, tetra-O-methylscutellarein, 5-O-demethylnobiletin, tangeretin, gardenin A, gardenin B	Dilution	HPLC-QqQ- MS/MS	[33]
Mandarin essential oil	**Five PMFs**: tangeretin, nobiletin, sinensetin, tetra-O-methyl scutellarein, heptamethoxyflavone	Dilution	HPLC-PDA-MS	[34]
Cold-pressed *Citrus* essential oils	**Nine Cs**: coumarin, meranzin hydrate, herniarin, citropten, meranzin, isomeranzin, aurapten, epoxyaurapten, 5-geranyloxy-7-methoxycoumarin; **19 FCs**: byakangelicin, 8-methoxypsoralen, psoralen, angelicin, oxypeucedanin hydrate, isopimpinellin, heraclenin, bergapten, isobergapten, byakangelicol, oxypeucedanin, imperatorin, phellopterin, cnidilin, isoimperatorin, epoxybergamottin, cnidicin, 8-geranyloxypsoralen, bergamottin; **seven PMFs**: sinensetin, nobiletin, tetra-O-methylscutellarein, tangeretin, 5-O-demethylnobiletin, gardenin A, gardenin B	Dilution	HPLC-PDA	[35]
Extra-virgin olive oils flavoured with aromatic plants	**Four Cs**: citropten, herniarin, meranzin, 5-geranyloxy-7-methoxycoumarin; **11 FCs**: bergamottin, bergapten, byakangelicol, cnidicin, cnidilin, isoimperatorin, isopimpinellin, oxypeucedanin, oxypeucedanin hydrate, phellopterin, 8-geranyloxypsoralen; **seven PMFs**: gardenin A, gardenin B, nobiletin, sinensetin, tangeretin, tetra-O-methylscutellarein, 5-O-demethylnobiletin	Dilution	HPLC-MS/MS	[36]
*Citrus* essential oils	**Two Cs**: citropten, herniarin; **16 FCs**: bergapten, psoralen, xanthotoxin, bergamottin, epoxybergamottin, byakangelicol, byakangelicin, isopimpinellin, imperatorin, isoimperatorin, oxypeucedanin, oxypeucedanin hydrate, heraclenin, phellopterin, 8-geranyloxypsoralen, angelicin	Dilution	UHPLC-TOF- MS	[37]
Bergamot essential oil	Untargeted compounds	Dilution	Ambient MS	[38]
*Citrus sinensis* oil	**Eight PMFs**: sinensetin, hexamethoxyflavone, tetramethyl-O-isoscutellarein, nobiletin, tetramethyl-O-scutellarein, heptamethoxyflavone, 5-demethylnobiletin, tangeretin	Dilution	HPLC-DAD	[39]
*Citrus* essential oils	**Ten Cs**: meranzin hydrate, herniarin, citropten, meranzin, isomeranzin, epoxyaurapten, osthol, 5-isopentenyloxy-7-methoxycoumarin, aurapten, 5-geranyloxy-7-methoxycoumarin; **15 FCs**: byakangelicin, oxypeucedanin hydrate, isopimpinellin, bergapten, byakangelicol, oxypeucedanin, isoimperatorin, imperatorin, cnidilin, epoxybergamottin, 5-(isopent-2′-eniloxy)-8-(2′,3′-epoxy)-isopentenyloxypsoralen, cnidicin, 8-geranyloxypsoralen, 5-geranyloxy-8-methoxypsoralen, bergamottin; **six PMFs**: sinensetin, hexamethoxyflavone, nobiletin, tetra-O-methylscutellarein, heptamethoxyflavone, tangeretin	Dilution	NanoUPLC- UV/EI-MS	[40]
*Cinnamomum cassia* Blume bark	**One C**: coumarin	UAE	HPLC-DAD	[41]
Cinnamon flavoring powders	**One C**: coumarin	UAE	HPLC-UV	[42]
Cinnamon powders and sticks	**One C**: coumarin	UAE	HPLC-UV	[43]
Cinnamon, tea, breakfast cereal, milk rice, cinnamon bun	**One C**: coumarin	UAE	HPTLC	[44]
*Citrus australasica* L. peel and pulp	**One C**: coumarin	UAE	HPLC-QTOF- MS/MS	[45]
Juices, beverages, jams, bakery products flavored with *Citrus* and cinnamon	**Nine Cs**: coumarin, aurapten, citropten, epoxyaurapten, herniarin, isomerazin, meranzin, meranzin hydrate, 5-geranyloxy-7-methoxycoumarin; **15 FCs**: 8-methoxypsoralen, bergamottin, bergapten, byakangelicin, byakangelicol, cnidicin, cnidilin, epoxybergamottin, isoimperatorin, isopimpinellin, oxypeucedanin, oxypeucedanin hydrate, phellopterin, 8-geranyloxypsoralen, psoralen; **four PMFs**: nobiletin, sinensetin, tangeretin, tetra-O-methylscutellarein	UAE LLE	SFC-QqQ- MS/MS	[46]
*Citrus*-flavored beverages and jams	**Eight Cs**: aurapten, citropten, epoxyaurapten, herniarin, isomeranzin, meranzin, meranzin hydrate, 5-geranyloxy-7-methoxycoumarin; **20 FCs**: angelicin, bergamottin, bergapten, byakangelicin, byakangelicol, cnidicin, cnidilin, epoxybergamottin, heraclenin, imperatorin, isobergapten, isoimperatorin, isopimpinellin, oxypeucedanin, oxypeucedanin hydrate, phellopterin, psoralen, trioxsalen, 8-geranyloxypsoralen, 8-methoxypsoralen; **Seven PMFs**: gardenin A, gardenin B, nobiletin, sinensetin, tangeretin, tetra-O-methylscutellarein, 5-O-demethylnobiletin	UAE LLE	HPLC-MS/MS	[47]
Pummelo fruits	**Four Cs**: umbelliferone, scoparone, limettin, isomeranzin; **eight FCs**: psoralen, bergaptol, xanthotoxin, bergapten, 6′,7′-dihydroxybergamottin, imperatorin, isoimperatorin, 6′,7′-epoxybergamottin; **eight PMFs**: eupatorin-5-methylether, sinensetin, 3′,4′,5,5′,6,7-heptamethoxyflavone, nobiletin, 5-hydroxy-7,8,4′-trimethoxyflavone, tangeretin, 5-hydroxy-3′,4′,7-trimethoxyflavone, 5-hydroxy-3,7,3′,4′-tetramethoxyflavone	UAE	UHPLC-QqQ- MS/MS	[48]
Satsuma mandarin peels and pulp	**Eight Cs**: umbelliferone, isomeranzin, scoparone, meranzin hydrate, limettin, scopoletin, aurapten, 5-geranyloxy-7-methoxycoumarin; **11 FCs**: bergaptol, psoralen, isopsoralen, xanthotoxin, bergapten, 6′,7′-dihydroxybergamottin, imperatorin, isoimperatorin, 6′,7′-epoxybergamottin, 8-geranyloxypsoralen, bergamottin; **nine PMFs**: 5-hydroxy-7,8,4′-trimethoxyflavone, eupatorin-5-methylether, 5,7,3′,4′-tetramethoxyflavone, 5,3′-dihydroxy-3,6,7,4′-tetramethoxyflavone, 5-demethylnobiletin, 3′,4′,5,5′,6,7-hexamethoxyflavone, sinensetin, tangeretin, nobiletin	UAE	HPLC-QqQ- MS	[49]
*Artemisia annua*	**Two Cs**: scopolin, scopoletin	UAE	HPLC-DAD	[50]
*Heracleum sphondylium* L. and *Aesculus hippocastanum* L.	**Six Cs**: coumarin, scoparone, isoscopoletin, esculin, esculetin, umbelliferone; **six FCs**: xanthotoxin, byakangelicin, isopimpinellin, bergapten, phellopterin, xanthotoxol	UAE	MEKC	[51]
Shiranuhi fruit and peels	**One PMF**: tetramethyl-O-scutellarein	SLE	MPLC	[53]
*Melilotus officinalis* L. and propolis	**Nine Cs**: esculin, daphnetin, fraxetin, umbelliferone, 4-methylumbelliferone, 4-hydroxycoumarin, scoparone, coumarin, herniarin	SLE	UHPLC-UV- FLD	[54]
Sweet clover herb, hay, and spoiled hay	**Three Cs**: dicumarol, coumarin and 4-hydroxycoumarin	SLE	HPLC-DAD	[55]
*Citrus* fruits	**One PMF**: nobiletin	SLE	HPLC-UV	[56]
*Citrus* peels	**Thirteen Cs**: trihydroxycoumarin hexoside, trihydroxycoumarin hexoside isomer, methoxy-trihydroxycoumarin hexoside, methoxy-trihydroxycoumarin hexoside isomer, methoxy-umbelliferone-hexoside, methoxy-trihydroxycoumarin hexoside isomer, dimethoxy-umbelliferone hexoside, benzyl-methyl-cyclohexanecarboxylateumbelliferone pentoside, umbelliferone, hydroxy-trimethoxy-methylchromen-4-one, allyloxy-dimethylcoumarin, aurapten, aurapten isomer; **one FC**: epoxybergamottin; **seven PMFs**: tetrahydroxy-dimethoxyflavone, dihydroxy-dimethoxyflavone, dihydroxy-trimethoxyflavone, dihydroxy-trimethoxyflavone isomer, dihydroxy-methoxyflavanone, dihydroxy-tetramethoxyflavone, hydroxy-pentamethoxyflavone	SLE	UPLC-QTOF- MS/MS	[57]
Vanilla extracts	**One C**: coumarin	SLE	MID-FTIR	[58]
*Citrus sinensis* peels	**Six PMFs**: 8-hydroxy-3,4′,5,6,7-pentamethoxyflavone, 5-hydroxy-6,7,8,3′,4′-pentamethoxyflavone, tangeretin, nobiletin, 3-methoxynobiletin, 7-hydroxy-3,5-dimethoxy-3, 4′-methylenedioxyflavone	SLE	TLC	[59]
*Citrus*-flavored beers	**Seven Cs**: aurapten, citropten, epoxyaurapten, herniarin, isomeranzin, meranzin hydrate, 5-geranyloxy-7-methoxycoumarin; **16 FCs**: angelicin, bergamottin, bergapten, byakangelicin, byakangelicol, cnidicin, cnidilin, epoxybergamottin, heraclenin, isoimperatorin, isopimpinellin, oxypeucedanin hydrate, phellopterin, psoralen, 8-methoxypsoralen, 8-geranyloxypsoralen; **seven PMFs**: gardenin A, gardenin B, nobiletin, sinensetin, tangeretin, tetra-O-methylscutellarein, 5-O-methylnobiletin	LLE	HPLC-MS/MS	[60]
*Citrus* and herbliquors	**Eight Cs**: coumarin, herniarin, meranzin, meranzin hydrate, citropten, epoxyaurapten, auraptene, 5-geranyloxy-7-methoxy-coumarin; **21 FCs**: 8-methoxypsoralen, byakangelicin, psoralen, oxypeucedanin hydrate, angelicin, isopimpinellin, heraclenin, oxypeucedanin, bergapten, byakangelicol, isobergapten, 6′,7′-dihydroxybergamottin, imperatorin, trioxalen, phellopterin, cnidilin, epoxybergamottin, isoimperatorin, cnidicin, 8-geranyloxypsoralen, bergamottin; **seven PMFs**: sinensetin, nobiletin, tetra-O-methylscutellarein, 5-O-demethylnobiletin, tangeretin, gardenin A, gardenin B	LLE	HPLC-QqQ- MS/MS	[61]
*Citrus* juices	**Five Cs**: scopoletin, citropten, meranzin, isomeranzin, osthol; **6 FCs**: bergaptol, bergapten, oxypeucedanin, 6′,7′-dihydroxybergamottin, epoxybergamottin, bergamottin; **four PMFs**: sinensetin, nobiletin, heptamethoxyflavone, tangeretin	SPE	HPLC-PDA- FLD	[62]
*Citrus* juices	**Twelve Cs**: scopoletin, umbelliferone, herniarin, meranzin, isomeranzin, meranzin hydrate, citropten, auraptenol, marmin, osthol, aurapten, 5-geranoxy-7-methoxycoumarin; **16 FCs**: heraclenol, bergaptol, oxypeucedanin hydrate, byakangelicin, bergapten, heraclenin, isosinensetin, byakangelicol, oxypeucedanin, 6′,7′-dihydroxybergamottin, imperatorin, phellopterin, isoimperatorin, 6′,7′-epoxybergamottin, 8-geranyloxypsoralen, bergamottin; **eight PMFs**: sinensetin, tetramethyl-O-isoscutellarein, nobiletin, tetramethyl-O-scutellarein, heptamethoxyflavone, tangeretin, 5-demethylnobiletin, 5-demethyltangeretin	SPE	HPLC-PDA- FLD	[63]
*Citrus reticulata* cv. Suavissima peels	**Three PMFs**: nobiletin, tangeretin, 5-demethylnobiletin	SPE	HSCCC	[64]
Sweet clover herb, hay, and spoiled hay	**One C**: dicoumarol	SPE	HPLC-DAD	[65]
Tokaj wine	**Six Cs**: esculin, coumarin, herniarin, 4-methylumbelliferone, scoparone, scopoletin	SPE	HPLC-DAD- FLD	[66]
Cinnamon foods and plants (lavender, chamomile, archangel)	**Three Cs**: coumarin, 7-hydroxycoumarin, 7-methoxycoumarin	SPE	HPLC-DAD	[67]
Soft drink, biscuits, sesame paste	**Six Cs**: coumarin, 7-methoxycoumarin, 7-methylcoumarin, 7-diethylaminocoumarin, pyranocoumarin, 3,3′-carbonylbis (7-diethylaminocoumarin)	SPE	HPLC-MS/MS	[68]
Tsoureki, cinnamon biscuit, panettone	**One C**: coumarin	SPE	HPLC-DAD	[69]
*Citrus paradisi* Macf. fruit and juice	**Seven FCs**: bergaptol, psoralen, 8-methoxypsoralen, bergapten, 6′,7′-dihydroxybergamottin, epoxybergamottin, bergamottin	QuEChERS	UPLC-MS/MS	[70]
Foods and beverages, with *Citrus*, figs, vegetables, herbs, and spices	**Seven FCs**: bergaptol, psoralen, 8-methoxypsoralen, bergapten, 6′,7′-dihydroxybergamottin, epoxybergamottin, bergamottin	QuEChERS	UPLC-MS/MS	[71]
Cinnamon bakery products	**One C**: coumarin	QuEChERS	GC-MS	[72]
*Helichrysum italicum* flowers	**One C**: scopoletin	SFE	HPLC-UV	[73]
*Citrus unshiu* peels	**One PMF**: nobiletin	SFE	HPLC-UV-Vis	[75]
*Citri reticulatae pericarpium*	**Three PMFs**: nobiletin, 3,5,6,7,8,3′,4′-heptamethoxyflavone, tangeretin	SFE	HPLC-DAD	[76]
*Pithecellobium dulce* bark	**One FC**: bergapten	MAE	HPLC-UV/Vis	[77]
*Cinnamomum verum* bark	**One C**: coumarin	Soxhlet extraction	UHPLC-QqQ- MS/MS	[78]
*Prangos pabularia* essential oil	**One C**: suberosin	Hydrodistillation with Clevenger	GC-FID GC-MS	[79]
*Geijera parviflora* leaves	**Three Cs**: osthol, scoparone, xanthyletin; **one FC**: isopsoralen	SLE	GC-MS	[80]
Cassia cinnamon, chamomile tea, Tokaj wines	**Seven Cs**: 6,7-dihydroxycoumarin, 7,8-dihydroxy-6-methoxycoumarin, 7-hydroxycoumarin, 7-hydroxy-4-methylcoumarin, 6,7-dimethoxycoumarin, coumarin, 7-methoxycoumarin	on-line MISPE	HPLC-DAD	[81]
*Matricaria chamomilla*	**Two Cs**: herniarin, umbelliferone	Maceration	HPLC-PDA	[82]
Lemon and persian lime	**Three Cs**: herniarin, citropten, 5 geranyloxy-7-methoxycoumarin; **eight FCs**: oxypeucedanin hydrate, isopimpinellin, bergapten, bergamottin, byakangelicol, oxypeucedanin, 8-geranyloxypsoralen, 5-geranyloxy-8-methoxypsoralen	UAE LLE	HPLC-DAD	[83]
*Angelica dahurica* roots	**Two Cs**: osthol, umbelliferone; **six FCs**: angelicin, imperatorin, xanthotoxin, isoimperatorin, oxypeucedanin, xanthotoxol	UAE	SFC-PDA	[84]
*Ammi visnaga* (L.) Lam. fruits	**Five Cs**: dihydrosamidin, visnadin, samidin, khellin, visnagin	UAE	SFC-PDA	[85]
*Cnidium monnieri* (L.) *Cusson* fruits	**One C**: osthol; **one FC**: imperatorin	SLE	SP-SFC- UV/Vis	[86]

C: coumarin; FC: furocoumarin; PMF: polymethoxyflavone; UAE: ultrasound-assisted extraction; LLE: liquid–liquid extraction; SLE: solid–liquid extraction; SPE: solid-phase extraction; MISPE: molecularly imprinted solid-phase extraction; MAE: microwave-assisted extraction; SFE: supercritical fluid extraction; QuEChERS: Quick, Easy, Cheap, Effective, Rugged, Safe; HPLC: high-performance liquid chromatography; GC: gas chromatography; SFC: supercritical fluid chromatography; TLC: thin-layer chromatography; MEKC: micellar electrokinetic capillary chromatography; MPLC: medium pressure liquid chromatography; HSCCC: high-speed counter current chromatography; DAD: diode array detector; PDA: photodiode array detector; FLD: fluorometer detector; FID: flame ionization detector; MS: mass spectrometer; HRMS: high-resolution mass spectrometer; QqQ: triple quadrupole mass spectrometer; TOF: time-of-flight mass spectrometer; FTIR: Fourier transform infrared spectroscopy.

## 3. Analytical Methods Used to Characterize OHCs

The presence of OHCs in a wide variety of plants and, consequently, in foodstuffs has promoted the development of numerous methods for the determination and quantification of these compounds. In the last eight years, several analytical methods have been developed to characterize OHCs, including gas chromatography (GC) coupled with flame ionization detector (FID) and MS, HPLC with UV, PDA, fluorometer (FL), and MS, and SFC with PDA and MS. Because of the high molecular weight of these compounds, the analytical technique mainly used for their determination is HPLC coupled with a PDA or MS detector [87]. However, each of these analytical approaches has benefits and drawbacks. Firstly, it is difficult to detect many OHCs simultaneously because of the fact that these compounds have different structures and polarities. Therefore, one of the aspects to take into account when selecting an analytical approach is the number and type of analytes to be determined. Secondly, an environmental assessment must be carried out. It is advisable to evaluate the amount and type of solvents used, analysis time, energy consumption, and equipment costs. Finally, the analytical technique employed for the characterization of OHCs should be repeatable, accurate, sensitive to low levels of OHCs concentration, and suitable for several food samples.

**Figure 3:**
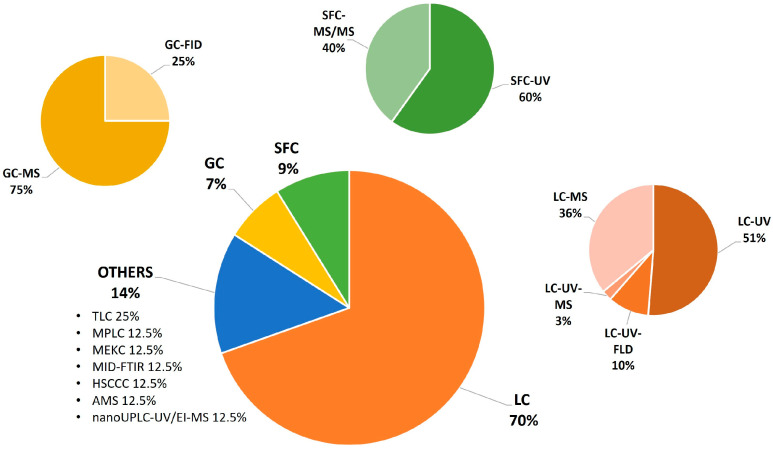
Type of analytical technique used for OHC analysis in foodstuffs (from 2016 to 2024).

A list of some of the methods used, reported in Figure 3, is presented in this section. Several parameters, such as chromatographic conditions, compounds detected, and environmental assessment, are explained for each method in order to facilitate the researchers’ choice of approach for their future work.

### 3.1. Gas Chromatography Methods

Gas chromatography can be used to determine some semi-volatile oxygen heterocyclic compounds, such as coumarin and furocoumarins with low molecular weight.

In 2016, Tabanca et al. [80] presented a detailed analysis of *Prangos pabularia* essential oil by means of GC-FID and GC-MS. One compound was identified as suberosin. In the same year, Sadgrove et al. [81] investigated the phytochemical diversity of *Geijera parviflora* leaves. Through the use of solvent extraction and GC-MS, several unknown and known coumarins (isopsoralen, scoparone, xanthyletine, osthole, and dehydrogeijerin) were identified, suggesting geographical variability in the plant’s chemical composition.

In 2017, Vetter et al. [72] used the QuEChERS sample preparation technique and GC-MS analysis to assess only the coumarin content in 14 different bakery products flavored with cinnamon.

As is evident from the mentioned publications, GC is limited to the determination of coumarin and a few other oxygenated heterocyclic compounds. To investigate the complete profile of these chemicals in plants and foods, further analytical methods are required.

### 3.2. Liquid Chromatography Methods

The most common methodologies, as shown in Figure 3, to determine OHCs in foods are based on HPLC with different detectors. HPLC has been shown to be a more efficient method for the chromatographic separation of these compounds, improving analytical sensitivity and resolution in a shorter retention time than other gas chromatographic techniques [3]. Among the detectors coupled to HPLC instrumentation, UV, DAD, and PDA were the most used because of (i) the presence of chromophores in the chemical structure of OHCs, (ii) the low cost of the detector, and (iii) ease of use. MS detectors were also used for OHC characterization, especially for OHCs present in trace amounts, or to improve the separation of some isomeric compounds.

Some of the research articles found in our literature survey were focused on the development of an analytical method only for the characterization of OHCs. On the other hand, some research papers reported the identification and quantification of OHCs together with other bioactive molecules, such as phenols, using the same HPLC analytical methodology [29,42,43,45,49,50,57,78]. For example, Cao et al. [42] and Pages-Rebull et al. [43] developed an HPLC-UV methodology to quantify key compounds in cinnamon, including coumarin, in order to discover adulterations. Fu et al. [50] and Santiago et al. [29] used an HPLC-DAD instrument to characterize coumarins, together with phenols and sesquiterpenes, in *Artemisia annua* L. and cachaça, respectively. HPLC coupled with a mass spectrometer detector was used by Aznar et al. [45], Fayek et al. [57], and Guo et al. [49] to investigate the content of bioactive compounds in *Citrus* fruits. The authors quantified various coumarins, furocoumarins, and polymethoxyflavones in the samples investigated. Ananthakrishnan et al. discussed the quantification of coumarin and other phenolic compounds in cinnamon samples from South India using an HPLC system coupled with a triple quadrupole mass spectrometer (HPLC-QqQ-MS) [78].

#### 3.2.1. HPLC Coupled with Spectrophotometric Detectors

Most of the HPLC analytical methods using a spectrophotometer as a detector were developed to identify and quantify only the OHCs considered as markers for monitoring authenticity (e.g., coumarin, bergapten, dicumarol) [41,55,65,67,77,81,82,83]. In all the research articles considered, the authors selected an octadecylsilyl (C18) stationary phase for the separation of molecules.

In 2017, Solaiman et al. [41] developed an HPLC-DAD method to determine the concentration of coumarin in the methanol extract of cinnamon bark (*Cinnamomum cassia* Blume). The researchers used a C18 column (250 mm × 4.6 mm, 5 μm) as the stationary phase. The analysis was in isocratic mode with a mobile phase composed of acetonitrile and 0.5% acetic acid in water (25:75) with a flow rate of 1 mL min^−1^. Coumarin was identified at a retention time of 10.4 min. The LoD and LoQ were 0.623 μg mL^−1^ and 1.889 μg mL^−1^, respectively. The mean concentration of coumarin in the cinnamon bark extract was found to be 916.71 mg kg^−1^. This information is important for determining compliance with the maximum limits set by food safety authorities. The same stationary phase was used by Katekhaye et al. [77] to determine the presence of bergapten in *Pithecellobium dulce* (Roxb.). The authors used acetonitrile and water (65:35, *v*/*v*) in isocratic mode, a flow rate of 1.0 mL min^−1^, and a UV wavelength of 266 nm. A C18 (250 mm × 4 mm, 5 μm) stationary phase was employed by Machynakova et al. [67,81] to quantify seven OHCs in complex food. Molecules were characterized using an HPLC-DAD system, and chromatographic separation was carried out with a mixture of acetonitrile/0.3% acetic acid (9:1, *v*/*v*) and acetonitrile as the mobile phase at a flow rate of 1 mL min^−1^.

A shorter C18 column (150 mm) was selected by Molnar et al. [82] to determine umbelliferone and herniarin concentrations in chamomile. Quantification of the two coumarins was carried out using water and methanol as the mobile phase at a flow rate of 1.0 mL min^−1^ with an HPLC-PDA instrument. Hroboňová and co-workers quantified dicoumarol [65] and coumarin, 4-hydroxycoumarin, and dicoumarol [55] from *Melilotus officinalis* (sweet clover) with a C18 (150 mm × 3.9 mm, 5 μm) column. The gradient elution was performed using methanol with 0.3% acetic acid and a 0.3% aqueous solution of acetic acid as the mobile phase. Jungen et al. [83] discussed the detection of adulteration in lemon and lime juices using coumarins and psoralens as chemical markers. The researchers used an HPLC-DAD method for quantitative analyses. Chromatographic separation was achieved using a C18 column (150 mm × 4.6 mm i.d., 2.6 μm). Eluents were tertiary mixtures of water/acetonitrile/tBME (A: 85/13/2, *v*/*v*/*v*) and acetonitrile/methanol/tBME (B: 65/30/5, *v*/*v*/*v*). The flow rate was 0.7 mL/min and the total run time was 50 min.

In 2018, a more polar stationary phase was selected by Li et al. [39] to characterize polymethoxyflavones present in orange peel oil. In this context, a C8 column (i.d. 4.6 mm × 150 mm, 2.7 µm particle) was used as stationary phase. The solvent consisted of 0.05% phosphoric acid/water (A), methanol (B), and 50% tetrahydrofuran/water (C) used in gradient mode, and the flow rate was 1 mL min^−1^ for 35 min. Eight polymethoxyflavones were identified in the sample.

In order to characterize a higher number of OHCs (including isomeric forms) in foods, it is necessary to use analytical instrumentation with higher performance in terms of selectivity. In 2019m Arigò and co-workers [35] developed an HPLC-PDA method combined with a linear retention index (LRI) approach and a UV-Vis library to characterize 35 OHCs in *Citrus* essential oils. The separation was carried out using a C18 column (50 mm × 4.6 mm, 2.7 μm) with water/methanol/tetrahydrofuran (85:10:5, *v*/*v*) as solvent A and methanol/tetrahydrofuran (95:5, *v*/*v*) as solvent B. The flow rate was 2 mL min^−1^ and the total analysis time was 14 min. The authors concluded that the LRI-based LC-PDA method, combined with the UV-Vis library, was a viable approach for screening and quantifying OHCs and even compounds with very similar spectra.

Another approach that can be used to improve the sensitivity and selectivity of the analytical method is the coupling of other detectors to a PDA. Several researchers developed chromatographic methods using serially coupled PDA and FLD [54,62,63,66]. Fluorescence detection provided high sensitivity, with detection limits in the ng mL^−1^ range for the coumarins.

In 2017, Machynakova et al. [54] developed a UHPLC method coupled with UV and FL detection for the simultaneous determination of nine coumarins (see Table 1). The separations were performed using a phenyl–hexyl silica-based analytical column (50 mm × 4.6 mm, 1.8 μm) under gradient elution with mobile phases consisting of acetonitrile and water (1:9 *v*/*v*) with 0.3% acetic acid (A) and acetonitrile (B). The total analysis time for the separation of the selected compounds was less than 6 min at a flow rate of 2.0 mL min^−1^. The limits of quantification were in the μg mL^−1^ range for UV detection and the ng mL^−1^ range for FL detection. The applicability of the method was demonstrated by analyzing plant (*Melilotus officinalis*) and propolis samples. In 2020, Hrobonova et al. [66] described the development and comparison between HPLC-DAD-FLD and fluorescence spectrometry methods for the determination of natural coumarins in Tokaj wine. The HPLC method was able to separate and quantify six coumarins using a C18 column (100 mm × 4.6 mm I.D., 5 μm) and a gradient elution with methanol/acetic acid (99/1 *v*/*v*) (A) and 1% aqueous solution of acetic acid (B). The analysis lasted 15 min at 1.0 mL min^−1^. DAD was used to monitor coumarin, while FLD was used for esculin, herniarin, 4-methylumbelliferone, scoparone, and scopoletin.

Li and co-workers developed two HPLC-PDA-FLD methods for determining OHCs in *Citrus* juices [62,63]. In the first method, the separation was performed on a C18 column (150 mm × 4.6 mm, 3.5 μm). The solvent consisted of 0.01% phosphoric acid/water (A), acetonitrile (B), and methanol (C) used in gradient mode at a flow rate of 1 mL min^−1^. The total analysis time was 55 min. The developed method provided a comprehensive approach for the analysis of sixteen bioactive *Citrus* compounds in a single run [62]. The second HPLC-PDA-FLD method developed by Li et al. allowed for the identification of a total of thirty-seven OHCs. The column, flow rate, and analysis time were the same as in the previous method. In addition to the mobile phase cited above, the researchers used a mixture of water/acetonitrile/tetrahydrofuran (55:20:25, *v*/*v*/*v*) as mobile phase D. Their work provided a detailed distribution pattern that helped to understand the relationship between *Citrus* juice intake and health outcomes [63].

#### 3.2.2. HPLC Coupled with Mass Spectrometer Detectors

Coumarins, furocoumarins, and polymethoxyflavones in foodstuffs can be better identified using LC with high-resolution mass spectrometry (HRMS) and tandem mass spectrometry (MS/MS) technologies, which are known to provide the best selectivity and sensitivity compared with traditional LC methods. These methodologies also allow for the chromatographic separation of isomers, ensuring a more accurate identification of the compounds present in the samples analyzed.

Regarding LC-HRMS, Masson et al. [37] developed a UHPLC/TOF-MS method to quantify 18 specific furocoumarins and coumarins in *Citrus* essential oils. The chromatographic separation was performed on a C18 stationary phase (100 mm × 2.1 mm, 1.8 μm). Solvent A was a mixture of water/methanol/tetrahydrofuran (85:10:5 *v*/*v*/*v*) with 0.1% of formic acid and 5 mM ammonium formate solution, while Solvent B was methanol/acetonitrile/tetrahydrofuran (65:30:5 *v*/*v*/*v*) with 0.1% of formic acid and 5 mM ammonium formate. The method successfully discriminated between *Citrus* species and detected adulteration with high sensitivity. Winstel et al. [30] investigated the role of oak-derived coumarins in contributing to the taste of wines and spirits during barrel aging. An LC-HRMS method was developed and validated to quantify six coumarins (Table 1) in various wines and spirits. The same stationary phase of the previous work was used with water containing 0.1% of formic acid (A) and acetonitrile with 0.1% of formic acid (B) as mobile phases. The flow rate was set at 600 μL/min for 12 min. An Orbitrap mass spectrometer equipped with a heated electrospray ionization (HESI II) probe was used. Their study provided new insights into the role of non-volatile oak-derived compounds, specifically coumarins, in shaping the sensory characteristics of aged wines and spirits.

Over the years, many researchers have developed HPLC-MS/MS methods to identify and quantify OHCs in *Citrus* fruits and *Citrus*-flavored foods [33,36,47,48,60,61,70]. In 2020, Zhao et al. [48] described the development of a rapid and sensitive UHPLC-QqQ-MS/MS method for the simultaneous qualitative and quantitative analysis of coumarins, furocoumarins, flavonoids, and phenolic acids in pummelo fruits. The column was C18 (2.1 × 100 mm, 1.8 µm), and the mobile phases consisted of 0.1% formic acid in water (A) and methanol (B). The detector was a QTRAP system equipped with a Turbo Spray Ion source, and it was used in multiple reaction monitoring (MRM) acquisition mode. Chromatographic conditions and MRM transitions were optimized to achieve good separation and accurate quantification of 47 analytes, including 13 groups of isomers, within a 13 min run time. The method presented low limits of detection and quantitation (0.014–1.50 μg L^−1^). The developed UHPLC-QqQ-MS/MS method combined high sensitivity, good selectivity, and short chromatographic run time, making it a versatile analytical tool for comprehensive profiling of these important secondary metabolites in pummelo fruits. Lee et al. [70] focused on developing an analytical method for identifying and quantifying furanocoumarins in grapefruit and their metabolites in human plasma and urine. Seven specific furanocoumarins were analyzed using UPLC-MS/MS. A C18 column (50 mm × 2.1 mm, 1.7 μm) was used for the analysis at a flow rate of 0.5 mL min^−1^ for 8 min. Mobile phases A and B were 0.1% formic acid in water and 0.1% formic acid in acetonitrile, respectively. The findings revealed that bergamottin and 6′,7′-dihydroxybergamottin were the predominant compounds in grapefruit and plasma, while bergaptol and 6′,7′-dihydroxybergamottin were the major compounds in urine. Arigò et al. [47] investigated the presence of OHCs in *Citrus* alcoholic and non-alcoholic beverages and jams using an HPLC-MS/MS method combined with an LRI system. The researchers used the same chromatographic condition of the previously validated HPLC-PDA method [35], using a triple quadrupole mass spectrometer via an atmospheric pressure chemical ionization (APCI) interface set in positive ionization mode. This analytical method was also used to analyze 16 commercial beer samples flavored with *Citrus* fruit and 18 extra-virgin olive oils flavored with aromatic and medicinal plants [36,60]. In 2024, Cafeo et al. [33,61] presented a fast and environmentally friendly HPLC-QqQ/MS method for analyzing OHCs in *Citrus*-flavored alcoholic and non-alcoholic beverages. Their method identified 36 OHCs in less than four minutes using water and ethanol as the mobile phase. The separations were achieved on a C18 column (50 mm × 2.1 mm, 2.7 µm). The HPLC system was hyphenated to a triple quadrupole mass spectrometer through an APCI interface operated in positive ionization mode. This approach enhanced the analysis of *Citrus* products, ensuring authenticity and safety with minimal environmental impact.

Other research articles focused on the development of the LC-MS/MS method to determine OHCs in foods and beverages known or suspected to contain these molecules [68,71]. Melough et al. [71] analyzed 29 food samples employing UPLC-MS/MS for detection after preparation using an SPE method. A C18 (50 mm × 2.1 mm, 1.7 μm) column was utilized for analyte separation. The mobile phase consisted of 0.1% formic acid in water (A) and 0.1% formic acid in acetonitrile (B). The total run time was 8 min with a constant flow rate of 0.5 mL min^−1^. The detection and quantification of the seven targeted analytes were performed in positive electrospray ionization mode (ESI+) with the MRM acquisition mode. Their findings showed that most foods contained multiple furocoumarins, with parsley, grapefruits, lime juice, grapefruit juice, and limes having the highest concentrations. Bergamottin, bergapten, and 6′7′-dihydroxybergamottin were the most commonly detected compounds. Their study provided valuable data for a more accurate estimation of dietary furocoumarin exposure and supported future epidemiological research on their health impacts. Nie et al. [68] presented a novel method for detecting coumarin and its derivatives in food matrices such as soft drinks, biscuits, and sesame paste. The researchers developed a urea-based magnetic adsorbent to perform the solid-phase extraction (MSPE) of OHCs. The analyses were carried out using an HPLC-MS/MS instrument. Six coumarins were separated by a C18 column (150 mm × 2.1 mm, 3.5 μm). The flow rate was set at 0.4 mL min^−1^. The researchers chose 0.1% formic acid aqueous solution (A) and acetonitrile (B) as the mobile phase at a flow rate of 0.4 mL min^−1^ for 15 min. MS/MS analysis was performed using a QTRAP mass spectrometer, with positive ion mode, in MRM. The method was environmentally friendly, requiring fewer organic reagents and offering high repeatability.

### 3.3. Supercritical Fluid Chromatographic Methods

SFC is an additional analytical method for the analysis of OHCs. By using a supercritical fluid, mainly CO_2_, as a mobile phase, SFC is able to combine the advantages of gas and liquid chromatography [84]. As SFC requires less solvent and less time for analysis than conventional chromatography, it is a more environmentally friendly analytical method. Although OHCs are excellent target molecules for the SFC approach (because of their low polarity), only a few scientific articles have been published in the last fifteen years on this topic [32,46,85,86,88].

Some of the first SFC methods for coumarins were developed with the use of spectrophotometric detectors to investigate a few selected OHCs in plants and fruits [85,86,88]. Pfeifer and co-workers [85] presented an SFC-PDA method for the determination of coumarins in the roots of *Angelica dahurica*. Eight compounds could be baseline-separated in 6 min using a Fluoro-Phenyl column (100 mm × 3.0 mm, 1.7 μm) with CO_2_, methanol, and diethylamine as the mobile phase. The method was applied to analyze coumarins in *Angelica dahurica* root samples. Imperatorin was the major coumarin (0.09–0.28%), followed by either isoimperatorin or oxypeucedanin. Winderl et al. [86] described an SFC-PDA method for the determination of five coumarins (Table 1) in *Ammi visnaga* fruits. The separation of these coumarins was achieved in less than 5 min using a C18 column (3.0 mm × 100 mm, 1.8 μm), CO_2_ as mobile phase A, and a mixture of methanol, acetonitrile, and 0.1% diethylamine as mobile phase B. The limits of detection were below 1.9 μg/mL for all compounds. The method allowed for the individual quantification of the previously co-eluting isomers dihydrosamidin and visnadin. Zhang et al. [88] developed a semi-preparative supercritical fluid chromatography method coupled with a UV/VIS detector (SP-SFC-UV/VIS) for the isolation and purification of osthol and imperatorin from *Fructus Cnidii*, a traditional Chinese medicinal plant. The optimized SP-SFC method used a YMC-Pack NH2 column (250 mm × 10.0 mm, 5 µm) and 3% ethanol as the modifier in the mobile phase at a flow rate of 20 mL min^−1^. Under these conditions, osthol and imperatorin were successfully isolated and purified from *Fructus Cnidii* extracts, with high purity confirmed by HPLC, NMR, and mass spectrometry analysis.

To the best of the authors’ knowledge, the first research article that combines the use of SFC with a tandem mass spectrometer for OHC analysis was published by Arigò and co-workers in 2022 [32]. The researchers developed an SFC-MS/MS analytical method for the analysis of twenty-eight OHCs in *Citrus* essential oils. Eight different stationary phases were evaluated, and a pentafluorophenyl column provided the best baseline separation of target analytes in under 8 min using CO_2_ and methanol as mobile phases. This method showed lower detection limits (0.0004–0.0470 mg kg^−1^) than those previously developed using PDA as a detector [87]. The validated method was then used to quantify the OHC profile of 26 *Citrus*-flavored food samples [46].

## 4. Conclusions

**Figure 4:**
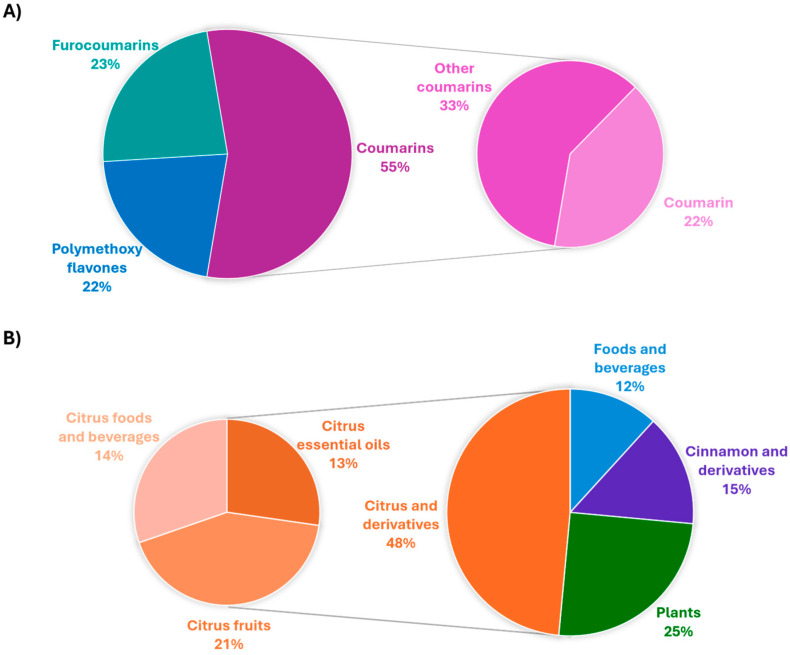
Schematic representation of (**A**) the types of OHCs and (**B**) the types of foodstuffs investigated from 2016 to 2024.

As can be seen in Figure 4A, in the last eight years, several authors have investigated all the OHC classes present in foodstuffs, with particular emphasis on coumarin. This can be attributed to the fact that coumarin is the sole molecule between OHCs for which strict regulations are in place regarding its presence in foodstuffs [12,13,15]. As reported in Figure 4B, more than 60% of the research articles considered in this review focused on the analysis of OHCs in cinnamon-flavored foods together with *Citrus*-based products. This is completely justified by the fact that cinnamon is the major dietary source of coumarin in the diet and that *Citrus* fruits are known to have all three classes of OHCs.

Our general overview of extraction techniques highlights the potential benefits of a purely dilution-based approach for the preparation of a sample, as it does not require the involvement of specialized personnel and is both time- and cost-effective. However, it is suitable only for liquid samples. For solid samples, several extraction methodologies have been compared. SFE has emerged as a highly effective alternative to conventional solvent extraction techniques to determine OHCs in foodstuffs. This approach is limited by the necessity of highly specialized personnel and expensive instrumentations. Conversely, ultrasound-assisted extraction represents an optimal choice among conventional techniques for solid samples, offering high sustainability, low cost, user-friendliness, and the ability to employ an appropriate solvent-to-sample ratio. In the process of validating an extraction procedure, it would be advantageous to assess extraction recovery. However, it is notable that only a limited number of authors have reported this aspect. The results of this survey indicate that the lowest recovery observed for OHCs in foodstuffs is 70.1%.

In terms of the chromatographic techniques used to identify and quantify OHCs, liquid chromatography is undoubtedly the technique of choice. Most of the scientific articles published on this subject consider the coupling of LC to spectrophotometric detectors since OHCs have chromophoric groups. This is because DAD and PDA are cheap and easy-to-use detectors. However, in order to separate isomeric compounds, analysis times need to be extended, and small percentages of acids or tetrahydrofuran should be added to the mobile phases. If the aim is to examine the complete profile of OHCs present in a sample, it is much more useful to couple more selective HPLC detectors, such as mass spectrometers. Indeed, with HPLC-MS or HPLC-MS/MS systems, it is possible to identify and quantify a greater number of OHCs, reducing both the analysis time and the volumes of solvents used. Thanks to the use of mass spectrometers, it is also possible to quantify the molecules of interest even if present in traces. The majority of HPLC-MS/MS methods developed and validated for the analysis of OHCs are capable of achieving a LoQ in the range of μg mL^−1^. Conversely, the drawbacks are associated with the expense of the apparatus, energy usage, and the requirement for appropriately skilled personnel. A “green” alternative is SFC. This is an advanced and expensive instrument that is not found in most quality control laboratories. However, further research using this analytical method is needed in the context of green chemistry.

In conclusion, if we want to imagine a future perspective in the extraction and analysis of OHCs in foodstuffs, the choice of developing miniaturized extraction techniques and the use of liquid chromatography that employs eco-friendly solvents could represent the most environmentally friendly choice without compromising analytical performance.

## Data Availability

No new data were created or analyzed in this study. Data sharing is not applicable to this article.
